# An Adjustable Smart Ring to Monitor Pulse Rate and Peripheral Blood Oxygen Saturation

**DOI:** 10.1007/s10439-025-03936-3

**Published:** 2025-12-10

**Authors:** Martina Montenegro, Andrea Aliverti, Alessandra Angelucci

**Affiliations:** Dipartimento di Elettronica, Informazione e Bioingegneria, Politecnico di Milano, Milan, Italy

**Keywords:** Smart ring, Wearable, Photoplethysmography, Pulse rate, Physical activity

## Abstract

**Purpose:**

Smart rings are emerging as a promising solution in the field of wearable devices, offering a compact and ergonomic solution for continuous physiological monitoring, yet one of their major limitations is that they are not adjustable, but rather come in different sizes. Another major gap in the literature is the lack of validation data during dynamic activities. This study presents the design, development, and validation under static and controlled-motion conditions of a research-grade adjustable smart ring for tracking pulse rate (PR) and peripheral blood oxygen saturation (SpO₂) using photoplethysmography.

**Methods:**

The device features a rigid-flex printed circuit board (PCB) optimized for finger placement, ensuring accurate sensor positioning, and adaptability to different sizes. Data transmission occurs via the ANT protocol, allowing real-time visualization on a mobile application. The smart ring was evaluated on 30 healthy volunteers (13 women and 17 men, mean age 27.5 ± 7.4 years, mean height 172.2 ± 9.0 cm, mean weight 68.1 ± 13.1 kg) through a multi-phase experimental protocol involving spontaneous breathing, apnea, and physical activity, with measurements compared against a gold-standard pulse oximeter.

**Results:**

Results demonstrate strong agreement in PR measurements both in static and dynamic conditions (*r* = 0.91, *p* < 0.001), while SpO₂ values exhibited an overestimation (mean bias = 1.04%).

**Conclusion:**

This work demonstrates the feasibility of an adjustable, research-grade smart ring based on a rigid–flex PCB for finger palmar PR and SpO₂ monitoring and quantifies its agreement with a clinical-grade fingertip oximeter in healthy adults in static and dynamic conditions. The device is conceived primarily as a hardware and measurement site platform that can be reused across ring sizes, while future work is needed to develop open signal-processing algorithms.

**Supplementary Information:**

The online version contains supplementary material available at 10.1007/s10439-025-03936-3.

## Introduction

The healthcare landscape is experiencing significant transformation, moving from traditional hospital-centric care to decentralized, patient-centered approaches. This shift is driven by factors such as rising healthcare costs, an aging population, and the need for continuous health monitoring [1]. Wearable devices have emerged as pivotal tools in this evolution, offering continuous, non-invasive, and real-time monitoring of various health parameters [2]. One of the most widely studied applications of wearable technology in healthcare is the continuous monitoring of vital signs [3], both in clinical settings [4] and remotely, thus enabling continuous outpatient monitoring.

Wearable devices are also instrumental in clinical applications including chronic disease management [5, 6], outcome and treatment effectiveness monitoring during clinical trials [7], functional capacity estimation [8], and wellbeing applications such as fitness tracking [9].

### Wearable Devices

Wearable devices can be found in different form factors, from the most common smartwatches [10] and wrist-worn trackers [11] to chest-worn patches [12], rings [13], earbuds [14], chest straps [15], bras or shirts [16], socks [17], gloves [18], necklaces [19], eyewear [20], headbands [21], belts [22], and intra-oral monitors [23]. These diverse wearable solutions enable monitoring of various physiological parameters. In this paper, we focus on devices that measure parameters related to peripheral vessel pulsation and its characteristics.

Cardiac parameters that can be extracted from wearable devices include heart rate (HR), heart rate variability (HRV), pulse rate (PR), pulse rate variability (PRV), and arterial blood pressure (ABP). Techniques to monitor cardiac parameters exploited by wearable devices include electrocardiography (ECG) [24], photoplethysmography (PPG) [8], seismocardiography (SCG) [25], ballistocardiography (BCG) [26], and phonocardiography (PCG) [27], with the 12-lead ECG considered the gold-standard method to monitor cardiac function.

Respiratory parameters that can be extracted from wearable devices include those related to breathing itself (respiratory rate, tidal volume, minute ventilation, inspiratory time, expiratory time, total time, duty cycle, mean inspiratory flow, and mean expiratory flow [28]), but also parameters that describe the content of gases in the blood [29], such as peripheral blood oxygen saturation (SpO_2_) and partial pressure of transcutaneous carbon dioxide (PtCO_2_) [30]. Different from the case of cardiac parameters, there is no gold-standard used for wearable monitoring of most respiratory parameters, but multiple strategies have been proposed in the literature [31]; the only exception to this is SpO_2_, which can be derived from PPG by means of pulse oximetry and is a well-established technique, as is described in the next section. Specifically, SpO_2_ is an immediate assessment of blood oxygen levels that does not require blood gas analysis since it is performed non-invasively with optical sensors. Monitoring SpO_2_ helps in the early detection of hypoxemia, which can increase morbidity and mortality in conditions such as chronic obstructive pulmonary disease (COPD) [32], acute respiratory infections (e.g., pneumonia [33], COVID-19 [34]), and sleep-disordered breathing [35]. Outside of the hospital, wearable monitoring of SpO₂ enables early detection of silent hypoxemia, tracking of exertional and nocturnal desaturation, home follow-up and exacerbation flagging in COPD [36], and screening for sleep-related hypoxemia [35].

SpO_2_ guides the management of oxygen therapy and ventilation in hospital settings and alerts medical staff in case of potential life-threatening declines, also in remote monitoring settings both in the case of chronic and acute diseases [37].

One general limitation of commercial wearable devices is that producers rarely disclose the details of the hardware and firmware, which limit the ability of researchers to use commercial devices outside of the functionalities envisioned by the manufacturers. Without prior knowledge of the hardware and firmware of the device, it is difficult to identify the sources of error and to refine the technology in order to enhance accuracy or reliability. Even if measuring accuracy is possible within proper experimental settings, the firmware running on a specific device can be updated by the company at any time, which would render any validation outdated. This lack of transparency poses a significant obstacle, especially in the healthcare field, where the validation and accuracy of methods are essential for safe and reliable use. For this reason, it remains relevant to propose research-grade wearable devices that can be tailored to the specific needs and research questions of the authors.

In this work, “research-grade” means full control of the hardware architecture, data flow, and firmware versioning, rather than to the use of fully open algorithms. Our approach consists of the selection of the sensor and firmware (either commercially available as in this case or custom-made), local, continuous access to data without external cloud services or closed APIs, version locking of firmware and settings for reproducibility, and possibility to substitute vendor algorithms in future iterations without modifying the hardware design. This level of control is typically missing in commercial black-box devices where architectures and algorithms are undisclosed and remotely updatable. Additionally, designing a research-grade device enables immediate integration with other devices in dedicated platforms [38–40] and study protocols, to meet the needs of the specific experimental campaign.

### Photoplethysmography in Wearable Devices

PPG is an optical technique used to detect changes in blood volume within a peripheral vascular bed. For basic blood volume pulse and PR monitoring, a single wavelength suffices. However, for SpO₂ estimation, two wavelengths are typically used—660 nm (red) and 940 nm (infrared)—because their differential absorption by oxy- and deoxyhemoglobin (HbO_2_ and Hb, respectively) enables oxygen saturation calculation [41]. Specifically, Hb and HbO_2_ absorb light differently at different wavelengths: at 660 nm the absorption of light is mainly due to Hb, while at 900 nm to HbO_2_ [42]. One significant advantage of PPG is its versatility in terms of body location. Notable measurement sites include the fingertip and wrist, commonly used in smartwatches and fitness trackers [43]. Other measurement sites include the arm, earlobe, oesophagus, forehead, thigh, leg, and ankle.

### Smart Rings

Smart rings, such as the Oura Ring [13, 44] and Galaxy Ring, are innovative wearable devices designed for health monitoring. Worn on the finger palmar surface, these rings utilize PPG technology to track vital metrics like PR, PRV, and SpO_2_.

The anatomical placement of smart rings on the finger palmar surface provides significant advantages over wrist-worn devices. The finger has a higher density of capillaries, resulting in improved blood perfusion and accurate PPG readings regardless of skin tone [45]. In contrast, wrist-worn devices often struggle with accuracy due to their placement on a less perfused area, which can lead to underestimations of SpO_2_ levels [38]. This issue is particularly pronounced in individuals with darker skin tones or during conditions of low perfusion, where wrist devices may be less reliable. While fingertip probes are known for their high accuracy, their bulkiness makes them impractical for everyday wear. Smart rings, however, maintain a user-friendly form factor that allows for unobtrusive and continuous health monitoring without the discomfort associated with larger devices. This balance of accuracy and usability positions smart rings as a compelling alternative to both wrist-worn devices and fingertip probes.

Several ring-type devices for physiological monitoring have been proposed in the literature [46]. Most devices are based on rigid printed circuit boards (PCBs), either flat or placed in contact with the finger where the sensors are [47–49], or curved. Flat PCBs do not reflect the finger’s shape, thus compromising real-life usability. One major limitation of devices based on rigid curved PCBs is that the electronics is not adjustable to different-sized fingers, therefore they must be produced in different sizes, increasing costs. More recent work has also investigated rigid–flex [50–52] or flexible PCB [53] ring oximeters to exploit high perfusion at the base of the finger, but some works do not report any validation results on humans (as Joo et al. [53]), and none take into account dynamic activities. Boukhayama et al. [50] enrolled 7 volunteers, and evaluated only PR and not SpO_2_. Martín-Escudero and Canabas [51] only enrolled 4 volunteer for a preliminary validation, and explicitly state they did not test the device under controlled-motion artifacts. Zhou et al. [52] collected data on 24 patients during surgical procedures. In summary, most reported prototypes rely on fixed-size PCBs that must be redesigned for each ring size, do not describe the hardware and data-access architecture in sufficient detail to serve as reusable research platforms, or they do not report validation data on humans during motion.

### Aim of the Work

Starting from the identified limitations of wearable devices, particularly wrist-worn PPG monitors, this study aims to design, develop, and validate a smart ring pulse oximeter capable of accurately monitoring PR and SpO₂ levels. Specifically, the study focuses on the comparison of two measurements performed with the same technique but on different sites, *i.e.*, the fingertip and the finger palmar surface. The device features a rigid-flex PCB tailored for ergonomic wearability and optimized sensor placement. Data are transmitted via the ANT protocol to a mobile application for real-time visualization. The validation process involves a multi-phase experimental protocol, including periods of normal breathing, apnea, and cycling, to explore lower ranges of SpO_2_ and higher ones of PR, with data compared against a gold-standard device. To best of our knowledge, there are no research works reporting validation data also during dynamic activities.

This paper details the design methodology, experimental procedures, and statistical analyses employed to evaluate the performance of the smart ring. By addressing challenges such as hardware design and validation on human volunteers in static and low-motion dynamic activities, this study provides a novel contribution to the advancement of wearable health monitoring technologies and underlines the different aspects that researchers must consider while designing validated research-grade rings.

## Materials and Methods

### Smart Ring Prototype

**Fig. 1:**
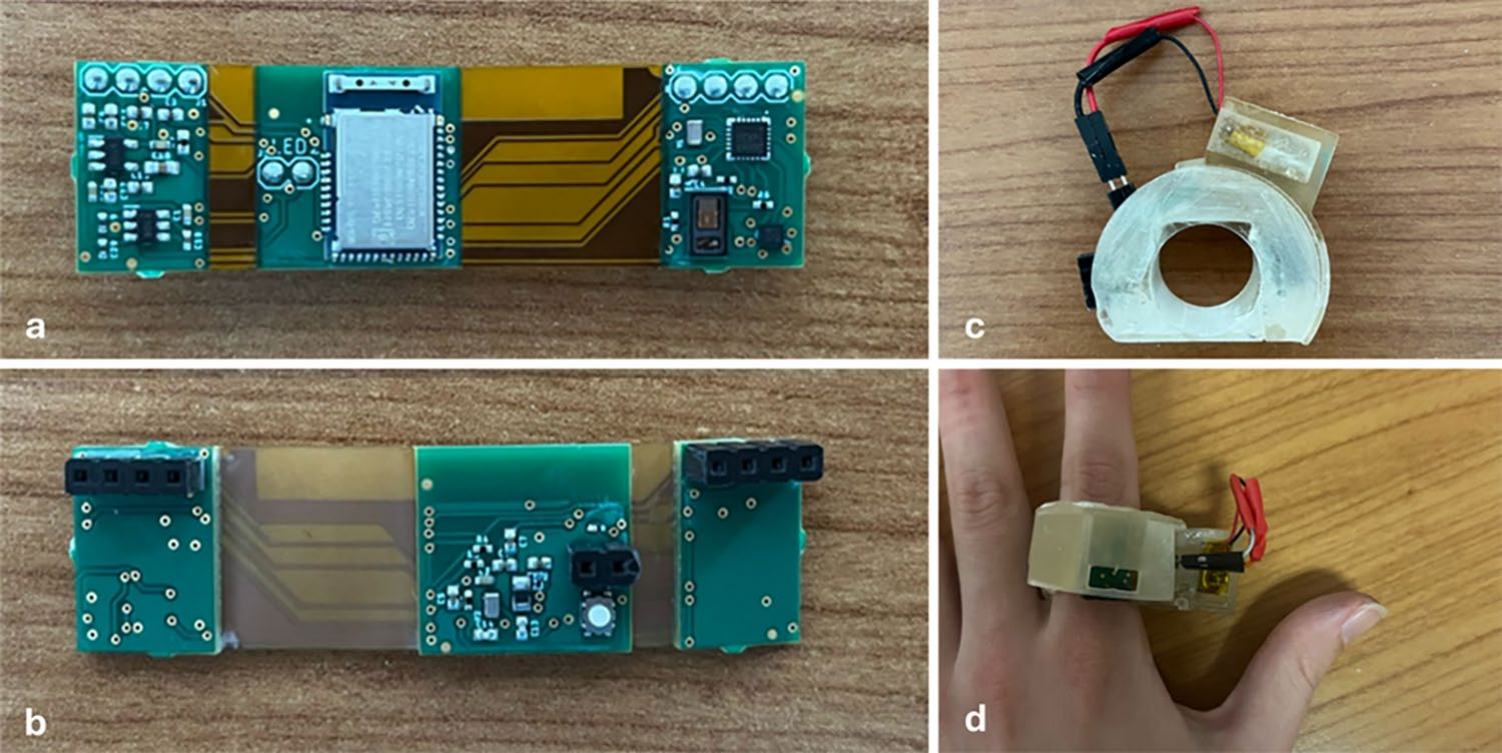
**a** Internal part of the PCB, applied on the finger palmar surface; **b** External part of the PCB; **c** PCB enclosed in a case and powered with a LiPo battery; **d** Smart ring when worn

The PCB of the smart ring prototype is shown in Fig. 1a (internal part, applied on the finger palmar surface) and 1b (external part), while Fig. 1c shows the PCB when enclosed in a 3D-printed case, and Fig. 1d represents the smart ring when worn.

The PCB dimensions are 61.47 mm (length) x 17.40 mm (height). The board is a rigid-flex PCB to accommodate the possibility to bend it around the finger and suit different sizes, thus it becomes adjustable to different sizes of the 3D plastic case, which in turn are customizable based on the size of the finger as explained later in the section.

The PCB is powered with a 3.7 V LiPo battery and contains three rigid panels (from left to right in Fig. 1a): one with a charge pump to raise the voltage up to 5 V, one with the microcontroller with an integrated 2.4 GHz antenna (nRF52832 [54]), and one with the two sensors (optical module MAX30101 [55] equipped with red, infrared, green LEDs, and accelerometer KX122-1037) and the associated sensor hub (MAX32664A [56]).

The device samples real-time SpO_2_ and PR data at 10 Hz, and raw data from the optical module are processed with the proprietary algorithms embedded in the sensor hub specifically designed for finger-based applications, and accelerometer data are used by sensor hub to remove motion artifacts [56]. The MAX30101 optical module integrates red (650–670 nm), infrared (870–900 nm), and green (530–545 nm) LEDs, although in this study only the red and infrared wavelengths were used. This configuration was selected because dual-wavelength (red + IR) PPG is required for SpO₂ estimation, and using the same spectral pair also allows direct comparison with the reference pulse oximeter. The MAX32664A sensor hub returns a single PR value. Moreover, this choice of configuration maintains continuity with previous laboratory prototypes based on identical hardware and algorithms, facilitating controlled validation of the measurement site rather than of wavelength-specific performance. Although green light is generally superior for motion-robust pulse-rate monitoring due to its shallower skin penetration and stronger pulsatile component, the main objective of this study was to validate the feasibility of finger palmar SpO₂ estimation using a dual-wavelength configuration comparable to standard pulse oximetry.

Processed data are transmitted to the smartphone, which has a dedicated app, at 1 Hz, by means of the ANT transmission protocol. The app was already described in other works [39, 57], and is designed to seamlessly integrate different wearable devices all transmitting data with ANT. In this case, it was only used to collect and save the data.

An ergonomic case was 3D-printed using a resin printer, to adjust the shape of PCB to the one of the finger (Fig. 1c) and guarantee adequate pressure of the optical module on the finger (Fig. 1d). The case has been 3D-printed in 3 sizes, i.e., with diameter of 19, 21, and 25 mm. No individualized case was printed for each participant. Adequate sensor pressure was achieved by allowing a slight protrusion of the sensor from the case on the lateral side of the finger. Only the best fitting ring size was used for each participant, while the other two sizes were not tested.

### Data Acquisition Protocol

The experimental protocol to test the smart ring prototype involved 30 healthy volunteers (13 women and 17 men, mean age 27.5 ± 7.4 years, mean height 172.2 ± 9.0 cm, mean weight 68.1 ± 13.1 kg), and was designed to assess whether the device could accurately detect valleys in SpO_2_ values caused by periods of apneas, and variations in PR induced by exercise. The protocol consisted of the following consecutive activities:

- 3 minutes of spontaneous breathing at rest (*REST*)
- 1 minute of breath holding (*APNEA*), followed by 2 minutes of spontaneous breathing at rest while recovering from the breath holding (*REC-APNEA*); this procedure was repeated three times
- 4 minutes of spontaneous breathing at rest (*REST*)
- 5 minutes of breathing while cycling on an exercise bike (*CYCLING*)
- 4 minutes of spontaneous breathing while recovering from the cycling phase (*REC-CYCLING*)

This protocol was chosen so that it would encompass both a physiological stimulus, induced by breath holding, and an exercise phase, as changes in parameters are expected in both cases but follow different mechanisms. Breath holding was a critical component, aimed at assessing the device’s ability to accurately track SpO_2_ during episodes of hypoxemia, which are more likely to occur when the device is used as an alarm system or for in-hospital monitoring. Since prolonged breath-holding could not be safely enforced outside of a clinical setting and without medical supervision, the procedure was repeated three times for shorter durations to induce transient drops in oxygen saturation. In contrast, the cycling phase was performed only once, as its longer duration was sufficient to elicit a noticeable increase in HR and PR.

All phases were conducted continuously without breaks. During *REST*, *APNEA*, *REC-APNEA* and *REC-CYCLING*, participants were seated with the tested hand resting on the lap; during *CYCLING*, participants pedaled on a stationary cycle ergometer while keeping the tested hand resting on the lap or on the handlebar to minimize extraneous arm movements. Stationary cycling was selected to safely elicit a broad physiological change in pulse rate under controlled laboratory conditions and to maintain a reliable fingertip reference measurement, in contrast with treadmill/running activities, which introduce larger motion artifacts [58]. To reduce placement variability, a standardized positioning procedure of the smart ring was followed for every trial (fixed finger, palmar location, and orientation relative to the proximal phalanx), and the case’s small protrusion maintained consistent contact. The smart ring was compared to a reference system, the WristOx2 3150 pulse oximeter by Nonin Medical Inc. (Plymouth, Minnesota, USA), which is FDA-cleared and has a measurement probe to be placed on the fingertip. The probe to perform the PPG measurement is applied on the fingertip exploits transmission pulse oximetry with red (660 nm) and IR (910 nm) LED transmission, while the smart ring is based on reflection pulse oximetry performed at the level of the finger palmar surface. The WristOx2 3150 uses its proprietary PureSAT® Artifact-free SpO_2_ technology to process the PPG signal, while the MAX32664A by Maxim Integrated sensor hub uses its proprietary firmware as explained in the previous subsection. Both companies do not fully disclose their algorithms.

Placing both devices on the same finger was not feasible due to overlapping footprints and the need for full contact at the palmar surface. Instead, adjacent fingers (index for the ring, middle for the fingertip probe) were used to ensure simultaneous, stable acquisition without interference. Prior studies have shown that SpO₂ values are consistent across different fingers in healthy volunteers [59], supporting the validity of this approach.

**Fig. 2:**
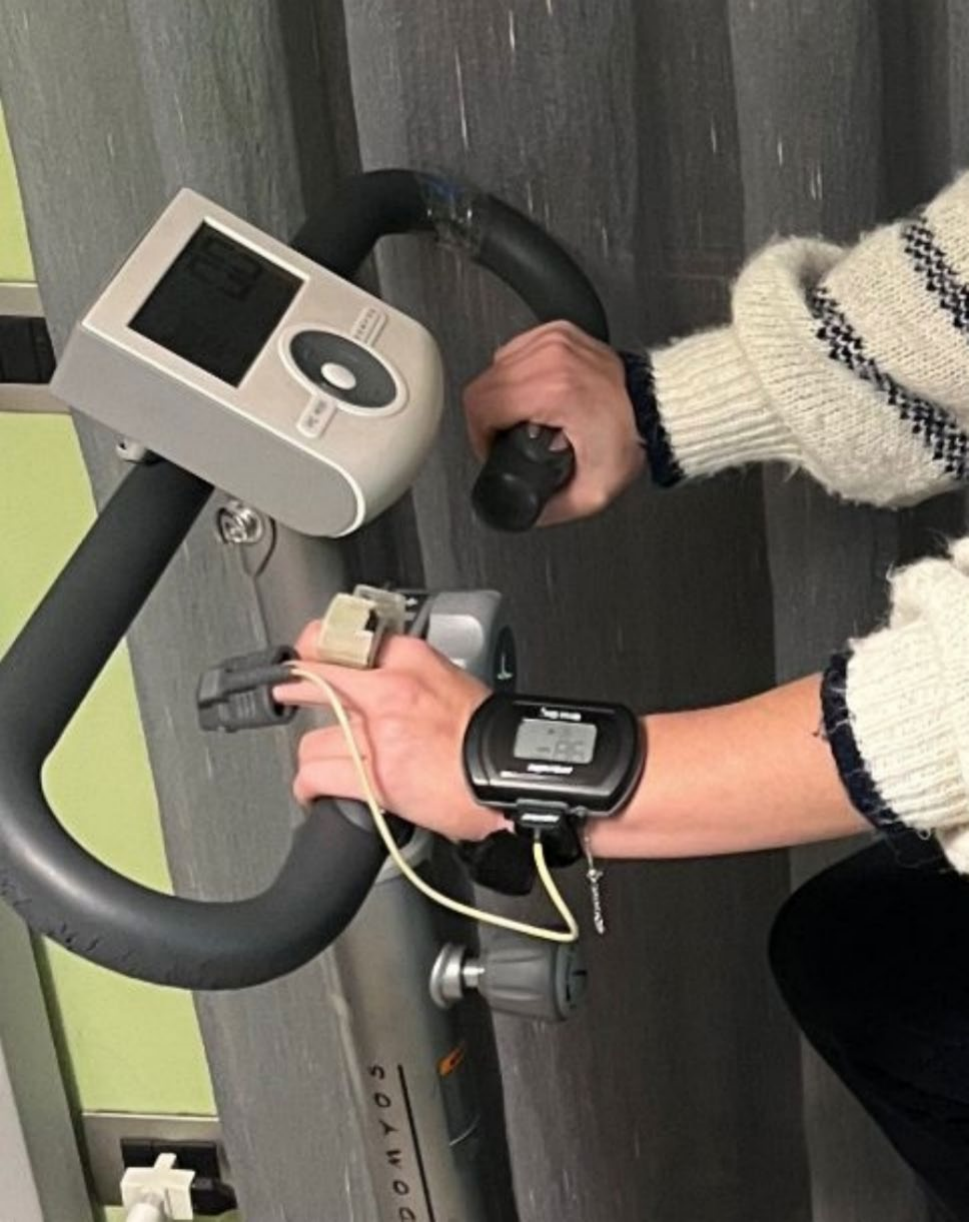
Study participant wearing the smart ring and the gold-standard Nonin WristOx2 wrist-worn pulse oximeter on her left hand. The smart ring is applied on the index finger, while the fingertip probe of the wrist-worn pulse oximeter is applied on the middle finger. During data collection, the hand rested on the lap/handlebar; the hand position shown here was posed solely for photographic clarity

Figure 2 shows one of the participants during the cycling phase.

The experimentation has been approved by the Ethical Committee of Politecnico di Milano (Protocol number: 34/2024; date of approval: 18/6/2024) and written informed consent has been obtained from all participants.

### Statistical Analysis

For each participant, PR and SpO₂ values were extracted from the smart ring, alongside corresponding timestamps of arrival of the data to the smartphone app. The reference system data were also parsed to retrieve PR and SpO₂ values. To achieve temporal synchronization between the smart ring and the reference system, missing timestamps were interpolated, the data detrended, and the optimal lag estimated via cross-correlation (or, in specific cases, a fixed lag applied), thereby enabling robust comparative analyses.

To improve data reliability, outliers were excluded using the z-score method. Measurements exceeding three standard deviations from the mean were considered outliers and removed.

The relationship between PR and SpO_2_ values obtained from the smart ring and from the reference system was visualized using scatterplots for each parameter, where the density of overlapping data points was computed using kernel density estimation (KDE) [60].

The linear agreement between the two systems was assessed both for PR and for SpO_2_ using Pearson’s correlation coefficient (r), with the corresponding p value computed to determine statistical significance.

Bland-Altman plots [61] were generated to assess agreement between the two devices by plotting the difference (smart ring—reference system) against the mean of both measurements. The mean difference (bias) and 95% limits of agreement (LoA) (mean difference ± 1.96 standard deviations) were calculated and displayed on the plot. KDE-based density mapping was applied to these plots, with darker colors indicating higher point concentration.

Histograms were included along the top and right axes of both scatterplots and Bland-Altman plots to represent the distributions of measurement values and differences, respectively.

In addition to analyzing the whole dataset together, the acquisitions were further segmented depending on the phase, and thus data were grouped with the 5 labels described in the experimental protocol: *REST* (before the apneas and before exercise), *APNEA* (during the apneas), *REC-APNEA* (recovery after each apnea), *CYCLING* (during the cycling phase, which is the only dynamic phase), *REC-CYCLING* (recovery after cycling). Pearson correlation coefficients and Bland–Altman analysis parameters were computed for each label, and the numerical results are reported in the Supplementary Material.

No direct quantification of motion artifacts (*e.g.*, artifact indices, signal-to-noise ratio) was performed, because neither device provides a standardized artifact quality output; thus, motion effects were addressed indirectly by reporting condition-specific results. A dedicated test–retest assessment of repeatability after deliberate ring removal and re-placement was not performed in this study.

## Results

This section reports a qualitative assessment of the traces, followed by the results on the agreement between the two measurements. Table 1 shows the main characteristics of the two parameters under study in all conditions, while Table S1 in the Supplementary Material reports a breakdown of the results divided by condition (*REST*, *APNEA*, *REC-APNEA*, *CYCLING*, and *REC-CYCLING*).

**Table 1:** Main characteristics of the two parameters under study (mean ± standard deviation, median [interquartile range])

Parameter	Device	Mean ± SD	Median [IQR]
PR [bpm]	Smart ring	79.68 ± 16.67	77 [22]
	Reference	79.41 ± 16.63	77 [22]
SpO_2_ [%]	Smart ring	97.12 ± 1.78	97 [2]
	Reference	96.08 ± 1.38	96 [2]

*SD* standard deviation; *IQR* interquartile range

### Qualitative Assessment of Traces

**Fig. 3:**
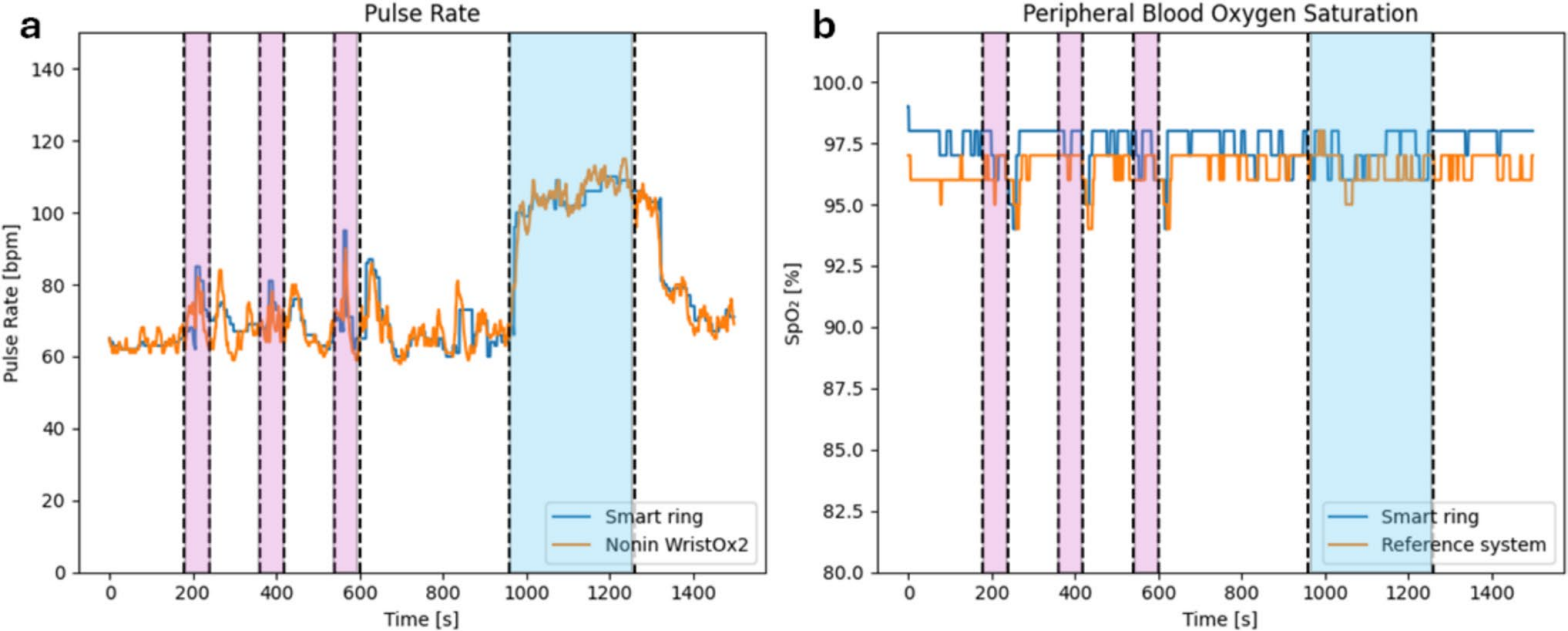
**a** Pulse rate during the whole test; **b** Peripheral blood oxygen saturation. Data obtained from the smart ring are shown in light blue, while data obtained from the Nonin pulse oximeter are shown in orange. Apnoeas are highlighted in pink, while the cycling phase is highlighted in light blue. All other samples are at rest or during recovery

Figure 3 shows the results obtained with the smart ring prototype compared to the outputs of the reference instrument, for one of the study participants (woman, 24 years old, 50 kg, 160 cm). Fig. 3a shows the PR and Fig. 3b shows the SPO2, and both figures highlight apnoeas (pink) and cycling (light blue).

The smart ring accurately tracks PR peaks after apnoeas and during cycling (Fig. 3a), and valleys in SpO_2_ after aponeas (Fig. 3b). During *APNEA* phases or immediately after (*REC-APNEA*), occasional transient spikes in PR values (up to ~110 bpm) were observed in some participants. These may reflect physiological arousal responses to breath-holding [62] or transient signal-quality fluctuations, but they were not systematic across all participants.

Since the study participants were all healthy volunteers, no valleys in SpO_2_ were expected during submaximal exercise. The smart rings displays a delay with respect to the Nonin WristOx2 in both measurements, and there is a consistent overestimation of SpO_2_ values (Fig. 3b). Reference SpO₂ values during non-apnea periods (*REST*, *CYCLING*, *REC-CYCLING*) averaged 96–97% (range 91–100%), which is within the expected range for healthy adults. Although slightly below an ideal 100%, such values are consistent with prior studies reporting resting SpO₂ of 95–98% in healthy individuals [34, 41, 46, 59].

Because stationary cycling limits upper-limb motion, the motion challenge applied to the optical sensors is lower than during ambulatory activities; this should be considered when interpreting agreement during the *CYCLING* segment.

### Agreement Between Measurements

**Fig. 4:**
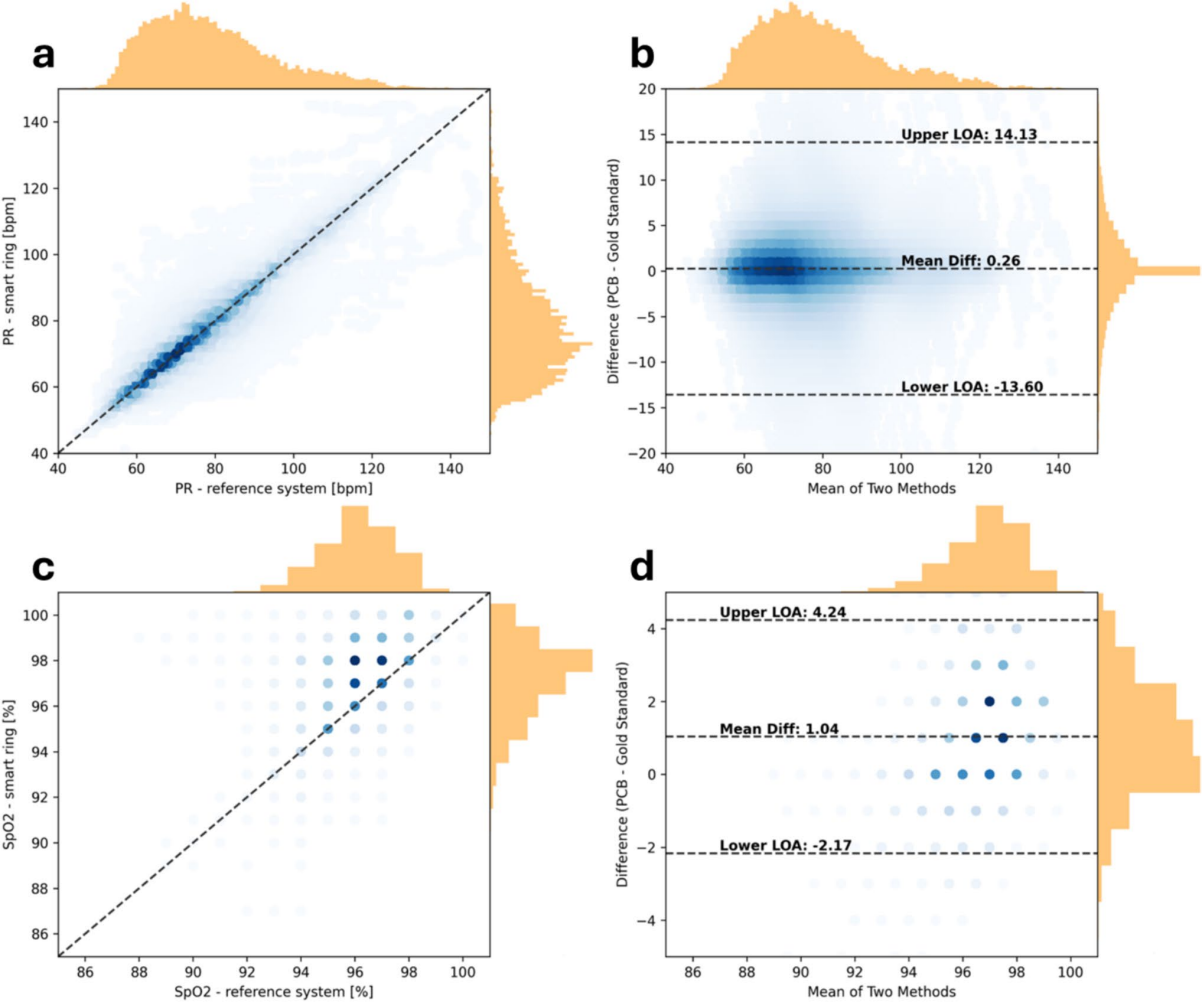
Comparison of smart ring and reference system, specifically: **a** Scatter plot of the PR values; **b** Bland-Altman plot of the PR values; **c** Scatter plot of the SpO_2_ values; **d** Bland-Altman plot of the SpO_2_ values

Figure 4 reports the agreement between the measurements obtained from the smart ring and those from the reference system, the Nonin WristOx2. Specifically, Fig. 4a shows the scatter plot of PR data obtained from the smart ring compared to those from the Nonin WristOx2, while Fig. 4b reports the Bland-Altman plot of the two measurement systems. Fig. 4c shows the scatter plot of SpO_2_ data obtained from the smart ring compared to those from the Nonin WristOx2, while Fig. 4d reports the Bland-Altman plot of the two measurement systems.

The Pearson’s correlation coefficient obtained in the case of PR is *r* = 0.91 (*p* < 0.001) and in the case of SpO_2_ is* r* = 0.49 (*p* < 0.001).

PR has a good correlation coefficient, and in the BA plot the mean difference shows that no consistent systematic errors are present (mean difference 0.26 bpm), and points are close to the bias without any trend. Meanwhile, the limits of agreement (LoAs) [− 13.60; 14.13] can be attributed to the high variation in the collected data, which include data collected in static and dynamic conditions, and are in line with other works using wearable devices in dynamic activities [38, 63, 64]. In fact, when only *REST* data are considered, LoAs are narrower: [− 10.09; + 10.55] bpm, while during the only dynamic activity (*CYCLING*) and its associated recovery phase (*REC-CYCLING*) LoAs are considerably larger, *i.e.*, [− 12.90; + 18.27] bpm and [− 18.54; + 13.53] bpm.

The high density of points around the identity line ($y=x$) suggests that most PR measurements from the smart ring closely match those from the reference system. Table S2 in the Supplementary Material reports a breakdown of the results divided by condition (*REST*, *APNEA*, *REC-APNEA*, *CYCLING*, and *REC-CYCLING*), in particular r and p value of the Pearson’s correlation and mean difference, upper and lower LoAs of the Bland-Altman analysis.

On the other hand, SpO_2_ displays a systematic bias: the smart ring tends to overestimate SpO_2_ values, and this is visible both in the scatterplot (Fig. 4c, where the highest density of points is above the identity line), and in the BA plot, where a mean difference of 1.04% between the two methods and LoAs in the range [− 2.17; 4.24] are depicted. Peripheral blood oxygen saturation results show an higher bias (absolute value) and narrower LoAs when compared to other works with similar measurement ranges and pulse oximetry sensors worn on the wrist, both in the case of commercial devices [65], and research-level devices developed in our laboratory [38].

The higher absolute value of the bias is understandable, since no calibration has been performed on the PCB prototype, while the fact that the smart ring overestimates the results of the reference system is opposite to what was observed in the other studies [38, 65].

## Discussion

The development and validation of the smart ring pulse oximeter presented in this study highlight the potential of a finger-based device to achieve accurate measurements of PR and SpO_2_ during dynamic activities while maintaining comfort and usability. Specifically, the measurement site in this case is the finger palmar surface, which demonstrates its ability to reliably detect the same parameters (PR and SpO_2_) that are generally detected at the fingertip when used in clinical settings. The adjustable hardware design, in which a single PCB is reused across different 3D-printed ring sizes, addresses the practical problem of finger-size variability without requiring multiple PCB designs and provides a reproducible research-grade platform for subsequent studies on smart-ring sensing, which is an important contribution to the existing literature. The validation data not only during static but also during low-motion dynamic activities are also a novel element of the presented work.

From the smart ring, it was possible to retrieve results comparable to fingertip devices, thus reinforcing the role of rings as valid alternatives to smartwatches. In fact, smart rings have gained increasing attention as compact wearable devices. A recent meta-analysis reported a mean bias of − 0.4 bpm for night PR estimation using ring-based photoplethysmography, though data heterogeneity remains high and SpO_2_ analyses are still limited [46]. A controlled hypoxia study has demonstrated that modern smart rings can achieve a root mean square error of approximately 2% in SpO_2_ estimation across different skin tones [45]. Similarly, ring-type oximeters have shown strong agreement with polysomnography-derived oxygen desaturation indices in sleep apnea assessment despite relatively wide limits of agreement [66]. These findings highlight that there is still the need to study these devices, validate them against gold-standard measurement systems, and continuously work towards their improvement. Within this context, the adjustable research-grade smart ring presented here contributes to the open exploration of sensor placement, contact pressure, and calibration strategies, bridging the gap between commercial rings (*e.g.*, Oura Ring, Viatom O₂Ring) and transparent, research-oriented designs suited for methodological validation and algorithm development.

Specifically, the smart ring’s performance was rigorously validated against the Nonin WristOx2 gold-standard pulse oximeter, using an experimental testing protocol, to explore wide ranges of both parameters that consisted in simultaneous acquisitions from the two devices. The protocol included periods of normal breathing, apnea, and physical activity to simulate a variety of physiological conditions. This allowed us to perform a feasibility study under static and low-motion conditions, including a physiological stimulus (apnea) and mild-intensity exercise performed with the hand kept still, thus minimizing motion at the sensor site.The Bland-Altman analysis revealed acceptable biases and LoAs for both PR and SpO_2_ measurements. Specifically, the device exhibited a PR bias of 0.26 beats per minute with LoAs of [− 13.60; 14.13], and an SpO_2_ bias of 1.04 % with LoAs of [− 2.17; 4.24]. These results suggest that the smart ring provides physiologically reliable data, despite the presence of a slight overestimation error for SpO_2_ values.

In the case of PR, the density of data points clearly highlights the accuracy of the measurements despite a relatively low precision. It is particularly important to highlight that the results in Fig. 4 are presented in an aggregated form, therefore they show a combination of rest, physiological stimuli (apnoeas), recovery from physiological stimuli, physical exercise (cycling), and recovery from physical exercise. The LoAs include a vast range of situations that can be encountered in activities of daily life.

The overestimation of SpO_2_ may stem from factors such as differences in perfusion between the base of the finger and the fingertip, as well as potential calibration limitations of the optical module. Notably, the MAX30101 optical module and its associated sensor hub algorithms are factory-calibrated and optimized by the manufacturer for fingertip applications, where tissue thickness, optical path length, and perfusion characteristics differ from those at the finger palmar surface. As a result, the calibration constants and signal-processing parameters embedded in the module may not perfectly match the optical properties of the palmar site, potentially contributing to the observed bias in SpO_2_ estimation. Future iterations of the device could address this issue by incorporating calibration procedures or solutions specifically designed for the finger palmar surface.

The smart ring employed red (650–670 nm) and infrared (870–900 nm) light, as these wavelengths are essential for SpO_2_ calculation based on the differential absorption of oxy- and deoxyhemoglobin. This configuration also ensured direct comparability with the gold-standard fingertip pulse oximeter, which uses the same pair of wavelengths. Conversely, several studies have demonstrated that green light (≈ 530 nm) can yield higher signal-to-noise ratios and reduced motion sensitivity for PR monitoring, particularly in wrist-worn devices where perfusion is lower [67]. However, since the finger palmar surface offers strong perfusion and minimal optical scattering, the red/IR configuration remained sufficient for robust PR detection while enabling simultaneous SpO₂ estimation. Future versions of the ring could incorporate multi-LED sequencing (green + red + IR) to evaluate wavelength-specific performance.

Compared to wrist-worn pulse oximeters, the smart ring offers distinct advantages. Finger sites provide higher perfusion than wrist sites and high-quality PPG signals [68], factors associated with stronger signal-to-noise ratio and potential resilience to motion; nonetheless, our protocol did not directly quantify artifacts nor include ambulatory motion, so superiority under high-motion conditions has not been demonstrated and remains to be tested. Still these factors likely contributed to the device’s consistent tracking of physiological trends, such as SpO_2_ valleys during apnea and PR peaks during exercise. Moreover, the ring’s rigid-flex PCB design and customizable 3D-printed casing provided an ergonomic fit, ensuring adequate pressure on the optical sensor for reliable measurements. From a manufacturing perspective, this strategy highlights the scalability of the design: the rigid-flex PCB is universally adaptable, while only the casing requires adjustment. In practical terms, this means that future large-scale production would not require personalized fabrication for every user, but rather a limited set of standardized sizes of the case, which is similar to existing smart rings like the Oura Ring and simplifies production while maintaining good fit and sensor contact.

However, the smart ring presented in this work still falls short of fully replacing clinical-grade devices. The LoAs for PR, while within acceptable ranges for consumer applications, remains relatively wide compared to stringent medical standards. A reference for medical-grade accuracy is the ANSI/AAMI EC13 (“Cardiac monitors, heart rate meters, and alarms”) standard by the American National Standards Institute, which requires an error of reading ≤  ± 10% or ≤  ± 5 bpm, whichever is greater [69]. In scientific literature, accuracy targets vary depending on the type of application (*e.g.*, at rest or during maximal exercise). In the work by Sartor et al. [70], wrist-worn PPG-based PR measurements were compared with chest-strap ECG-based HR, and the results were considered positive with a mean absolute error ≤ 3 bpm and limits of agreement within ± 15 bpm. Cadmus-Bertram et al. [71] report LoAs of PR measurements compared to ECG-based HR measurements during resting conditions in multiple devices, specifically [−4.1; + 4.5] bpm for Surge Fitbit, [−17.1; + 22.6] bpm for Basis Peak, [−10.5; + 4.5 bpm] for Fitbit Charge, and [−7.8; + 9.9]. Gillinov et al. [72] tested different wrist-worn PR monitors at rest and during exercise at light, moderate, and vigorous intensity, compared them to an ECG-based chest strap, and found that accuracy depends on exercise type. In our study, the Bland–Altman analysis showed a mean difference of only 0.26 bpm, and LoA of [−13.60; + 14.13] bpm not only at rest but also during moderate exercise, while comparing the smart ring to a medical-grade PPG-based PR monitor. These results fall well within the accuracy thresholds proposed by other authors, supporting the reliability of the device for PR measurement under the tested conditions.

Some points need to be addressed in future studies to carry out a more comprehensive experimental protocol. Evaluation under motion-intensive (‘dynamic’ in the PPG sense) conditions and activities inducing substantial finger movement will be part of future work, and such activities are expected to be highly affected by motion artifacts at the measurement site. This study did not include walking nor running or deliberately induced hand/arm motion; thus, it under-represents real-world motion artifacts. Neither device provided a standardized artifact index, precluding direct artifact quantification. Future studies will include ambulatory protocols (walking/running) with synchronized tri-axial acceleration, extraction of signal-quality metrics, and evaluation of accelerometry-assisted artifact suppression. Ring placement variability (micro-position, orientation, and contact force) can affect PPG waveform amplitude and quality; this is well-recognized for PPG across body sites and contact conditions [73, 74]. Yet, we did not perform a re-placement repeatability test here. Future work will quantify intra-site repeatability by controlled removal and re-placement, and contact force modulation.

Additionally, it was not possible to reach low levels of SpO_2_ with this protocol, as only healthy volunteers participated in the trial, and there was no physiological stimulus to induce significant desaturation. The use of reflection-mode photoplethysmography, rather than the transmission-mode employed by the reference oximeter, may partly explain the smart ring overestimation of SpO_2_. Reflection geometry involves a shorter optical path and is more sensitive to variations in tissue composition, perfusion, and contact pressure, which can alter the red/IR absorption ratio used for SpO_2_ estimation [67]. In contrast, transmission-based sensing measures light passing entirely through the finger, providing a more uniform optical path. Refinements to the sensor positioning and light path optimization could improve accuracy in future designs, and both dedicated protocols for desaturation and validation on patients are needed to assess the performance of the smart ring on a broad range of SpO_2_ values.

The compact design of the smart ring imposed constraints on component selection and integration, yet future developments will have to further reduce dimensions to improve usability during activities of daily life. For instance, the integrated microcontroller and RF antenna module (MDBT42Q) limited further miniaturization due to their footprint, and this limitation could be overcome using the single components (*i.e.*, only the microcontroller instead of the integrated module) and designing a dedicated antenna. Additionally, while the 3D-printed casing provided adaptability to different finger sizes, it introduced potential issues with light refraction due to its transparent resin material. A switch to opaque or black materials (*e.g.*, a plastic material like PLA) could mitigate this problem, enhancing measurement consistency.

Battery life and reusability were addressed through the inclusion of a rechargeable LiPo battery. However, the implementation of a curved battery could further improve the ring’s comfort and usability. Future iterations might also explore energy harvesting techniques to extend operational time without increasing the device size.

In this prototype, the proprietary algorithms embedded in the MAX32664A sensor hub were intentionally used to ensure reproducibility and comparability with previous PPG-based devices developed in our laboratory [38–40] and with commercial pulse oximeters employing the same hardware and sensor hub. This strategy allowed us to specifically validate the custom-made hardware configuration and measurement site, independently from the algorithmic layer. It must be noted that Maxim Integrated is a leader in the market of PPG sensors and sensor hubs and, while it does not fully disclose the proprietary algorithm, provides extensive documentation on their products including the performance of the algorithms in the MAX32664 sensor hub, including some embedded development software that can be freely downloaded [56]. Maxim Integrated devices are largely used in commercial wearable devices and research projects alike and easily available to other researchers that want to reproduce the presented work. Nevertheless, relying on manufacturer-specific algorithms entails certain drawbacks, such as the lack of transparency in the signal-processing steps. While this remains a limitation of the present work, it is mitigated by the fact that in this case the firmware version was locked, differently from the case of commercial devices that act as a black-box and are subject to producers upgrading the firmware without possibility to track the changes. Hardware control, deterministic configurations, local logging, and version stability enable rigorous synchronization and repeatable experiments to performed a controlled validation, and this is made possible by our research-grade device. Commercial wearables typically lack this degree of control and can be silently altered by server-side updates.

Future iterations of the smart ring will therefore include custom-developed or open-source [75] signal-processing algorithms for raw PPG data, enabling adaptive calibration, real-time signal-quality assessment, and motion-artifact suppression tailored to the palmar-finger configuration. This can be done using the same hardware as the MAX32664 sensor hub also allows to extract raw data, as per its datasheet [56].

It is noteworthy to highlight that the presented smart ring was integrated into an existing mobile application for data visualization, collection and storage [39], and this enables to obtain simultaneous measurements from other integrated devices that support the ANT transmission protocol synchronization between the integrated devices would also remove the need for a-posteriori signal alignment to analyze data.

The results obtained in this study suggest that the smart ring is well-suited for continuous monitoring in non-critical environments, such as fitness tracking and wellness applications. Its ergonomic design and acceptable accuracy make it an attractive option for long-term use in consumer health markets, after proper miniaturization. However, to achieve medical-grade reliability, additional calibration and validation against a broader range of gold-standards, during different activities (such as walking and running), and in response to physiological stimuli such as induced desaturation are necessary.

## Supplementary Information

Below is the link to the electronic supplementary material.

Supplementary file1 (DOCX 299 kb)

## Data Availability

The data that support the findings of this study are available from the corresponding author upon reasonable request.

## Abbreviations

ABP: Arterial blood pressure
BCG: Ballistocardiography
ECG: Electrocardiography
HR: Heart rate
HRV: Heart rate variability
PCB: Printed circuit board
PCG: Phonocardiography
PPG: Photoplethysmography
PR: Pulse rate
PRV: Pulse rate variability
SCG: Seismocardiography
SpO_2_: Peripheral blood oxygen saturation
