# Supplementary material for: An Adjustable Smart Ring to Monitor Pulse Rate and Peripheral Blood Oxygen Saturation
Source: Ann Biomed Eng. 2025 Dec 10;54(3):885–97. doi: 10.1007/s10439-025-03936-3 (PMC12960368; doi:10.1007/s10439-025-03936-3)
Supplement: Supplementary file 1 — Supplementary file1 (DOCX 299 kb) [file 10439_2025_3936_MOESM1_ESM.docx]

# An Adjustable Smart Ring to Monitor Pulse Rate and Peripheral Blood Oxygen Saturation

Martina Montenegro^1^, Andrea Aliverti^1^, Alessandra Angelucci^1,^*

^1^ Dipartimento di Elettronica, Informazione e Bioingegneria, Politecnico di Milano, Milan, Italy

* Corresponding author: [alessandra.angelucci@polimi.it](mailto:alessandra.angelucci@polimi.it); ORCID: 0000-0001-8266-9346

# Supplementary Material

| **Phase** | **Parameter** | **Device** | **Mean ± SD** | **Median [IQR]** |
| --- | --- | --- | --- | --- |
| *REST* | PR [bpm] | Smart ring | 72.89 ± 10.77 | 72 [17] |
|  |  | Reference | 72.66 ± 10.67 | 72 [17] |
|  | SpO_2_ [%] | Smart ring | 97.02 ± 1.75 | 97 [2] |
|  |  | Reference | 96.06 ± 1.35 | 96 [2] |
| *APNEA* | PR [bpm] | Smart ring | 73.11 ± 11.10 | 73 [15] |
|  |  | Reference | 73.56 ± 11.03 | 73 [15] |
|  | SpO_2_ [%] | Smart ring | 97.20 ± 1.95 | 98 [2] |
|  |  | Reference | 96.22 ± 1.46 | 96 [2] |
| *REC-APNEA* | PR [bpm] | Smart ring | 72.54 ± 10.85 | 71 [17] |
|  |  | Reference | 72.05 ± 10.79 | 71 [17] |
|  | SpO_2_ [%] | Smart ring | 97.23 ± 1.84 | 98 [2] |
|  |  | Reference | 96.19 ± 1.45 | 96 [1] |
| *CYCLING* | PR [bpm] | Smart ring | 96.95 ± 17.01 | 98 [24] |
|  |  | Reference | 94.26 ± 17.20 | 95 [26] |
|  | SpO_2_ [%] | Smart ring | 96.94 ± 1.73 | 97 [2] |
|  |  | Reference | 95.92 ± 1.40 | 96 [2] |
| *REC-CYCLING* | PR [bpm] | Smart ring | 83.90 ± 17.15 | 80 [23] |
|  |  | Reference | 86.40 ± 18.80 | 83 [25] |
|  | SpO_2_ [%] | Smart ring | 97.26 ± 1.61 | 98 [2] |
|  |  | Reference | 96.06 ± 1.16 | 96 [2] |

**Table S1.** Main characteristics of PR and SpO_2_ during each phase of the trial (mean ± standard deviation, median [interquartile range]. SD = standard deviation; IQR = interquartile range. The five phases are *REST*, *APNEA*, *REC-APNEA*, *CYCLING*, and *REC-CYCLING*.

| **Phase** | **Parameter** | **Pearson’s correlation** | | **Bland Altman Analysis** | | |
| --- | --- | --- | --- | --- | --- | --- |
|  |  | **r** | **p-value** | **Mean of differences** | **Upper LoA** | **Lower LoA** |
| *REST* | PR [bpm] | 0.88 | p < 0.001 | 0.23 | 10.55 | -10.09 |
|  | SpO_2_ [%] | 0.49 | p < 0.001 | 0.96 | 4.12 | -2.20 |
| *APNEA* | PR [bpm] | 0.79 | p < 0.001 | -0.45 | 13.57 | -14.46 |
|  | SpO_2_ [%] | 0.44 | p < 0.001 | 0.97 | 4.60 | -2.65 |
| *REC-APNEA* | PR [bpm] | 0.83 | p < 0.001 | 0.49 | 12.77 | -11.80 |
|  | SpO_2_ [%] | 0.47 | p < 0.001 | 1.04 | 4.41 | -2.33 |
| *CYCLING* | PR [bpm] | 0.89 | p < 0.001 | 2.68 | 18.27 | -12.90 |
|  | SpO_2_ [%] | 0.57 | p < 0.001 | 1.02 | 3.93 | -1.88 |
| *REC-CYCLING* | PR [bpm] | 0.90 | p < 0.001 | -2.51 | 13.53 | -18.54 |
|  | SpO_2_ [%] | 0.42 | p < 0.001 | 1.20 | 4.20 | -1.81 |

**Table S2.** Results of Pearson’s correlation and of the Bland-Altman analysis of PR and SpO_2_ during each phase of the trial. The five phases are *REST*, *APNEA*, *REC-APNEA*, *CYCLING*, and *REC-CYCLING*.
